# CORE-PH: A 3-Component Score for Long-Term Mortality Risk in a Pulmonary Arterial Hypertension Cohort

**DOI:** 10.3390/diagnostics16183057

**Published:** 2026-09-20

**Authors:** Çetin Alak, Furkan Fatih Yurdalan, Fazıl Çağrı Hunutlu, Zeynep Kumral, Ayşe Nilgün Kara, Ahmed Faruk Koç, Şeyda Günay Polatkan, Dilek Yeşilbursa

**Affiliations:** 1 Department of Cardiology, Faculty of Medicine, Bursa Uludag University, Bursa 16059, Türkiye; ffyurdalan@gmail.com (F.F.Y.); aysenilgunkara@uludag.edu.tr (A.N.K.); seydagunay@uludag.edu.tr (Ş.G.P.); dilekyb@uludag.edu.tr (D.Y.); 2 Division of Hematology, Department of Internal Medicine, Bursa Sehir Training and Research Hospital, University of Health Sciences, Bursa 16009, Türkiye; fazilhunutlu@gmail.com; 3 Department of Cardiology, Unye State Hospital, Ordu 52300, Türkiye; zeynepkumral@gmail.com; 4 Department of Family Medicine, Faculty of Medicine, Bursa Uludag University, Bursa 16059, Türkiye; ahmedkoc@uludag.edu.tr

**Keywords:** pulmonary arterial hypertension, risk stratification, right ventricular–pulmonary arterial coupling, mixed venous oxygen saturation, Prognostic Nutritional Index, mortality

## Abstract

**Background/Objectives**: Pulmonary arterial hypertension (PAH) risk tools do not jointly capture right ventricular adaptation, systemic oxygen balance, and nutritional reserve. We developed the three-component CORE-PH score and assessed its association with long-term mortality. **Methods**: This retrospective, single-center study included 105 treatment-naïve adults with group 1 PAH or group 4 chronic thromboembolic pulmonary hypertension who underwent diagnostic right heart catheterization in 2011–2025. One point each was assigned for Prognostic Nutritional Index ≤ 47.7, TAPSE/sPAP ≤ 0.4632 mm/mmHg, and mixed venous oxygen saturation ≤ 68.1%. Mortality associations were assessed using Cox regression. Discrimination was compared with COMPERA 2.0 and REVEAL Lite 2 using time-dependent AUCs at 12, 36, and 60 months. Bootstrap validation repeated threshold selection and score reconstruction. **Results**: Of 105 patients, 98 (93.3%) had PAH and 51 died during a median follow-up of 70.0 months. Each CORE-PH point was associated with greater mortality after adjustment for age and functional class (hazard ratio, 2.645; 95% confidence interval, 1.823–3.837; *p* < 0.001). The association persisted in the contemporary-definition sensitivity cohort (*n* = 84). Time-dependent AUCs were 0.779, 0.779, and 0.817 at 12, 36, and 60 months. AUCs were higher than those of COMPERA 2.0 at 36 and 60 months, while differences versus REVEAL Lite 2 were not statistically significant. The optimism-corrected C-index was 0.719, and the optimism-corrected calibration slope was 0.888. **Conclusions**: CORE-PH identified a mortality gradient in this predominantly PAH cohort. External validation is required before clinical use; applicability to other forms of precapillary pulmonary hypertension remains uncertain.

## 1. Introduction

Pulmonary arterial hypertension (PAH) is a progressive pulmonary vascular disease characterized by increasing pulmonary vascular resistance, right ventricular failure, and premature death. Despite substantial advances in PAH-specific therapies, clinical trajectories and long-term outcomes remain heterogeneous, making accurate risk stratification central to both initial treatment selection and subsequent therapeutic escalation. Contemporary guidelines recommend multidimensional risk assessment at diagnosis and during follow-up, incorporating clinical status, exercise capacity, circulating biomarkers, cardiac imaging, and invasive hemodynamics. Achieving and maintaining a low-risk profile is a major treatment objective, whereas intermediate-high or high-risk status may prompt earlier treatment intensification and consideration of advanced therapies [1].

Several validated tools have been developed to facilitate risk assessment in routine clinical practice. REVEAL Lite 2 is an abbreviated, entirely noninvasive score based on World Health Organization functional class (WHO-FC), systolic blood pressure, heart rate, six-minute walk distance (6MWD), BNP or N-terminal pro-B-type natriuretic peptide (NT-proBNP), and renal function [2]. COMPERA 2.0 uses WHO-FC, 6MWD, and BNP or NT-proBNP to classify patients into low-, intermediate-low-, intermediate-high-, and high-risk strata, thereby improving prognostic separation within the heterogeneous intermediate-risk population [3]. Although these instruments were originally developed for partly different time horizons and clinical settings, both have demonstrated prognostic value at baseline and during longitudinal assessment and represent clinically relevant benchmarks for PAH risk stratification [2,3,4]. Nevertheless, these simplified tools predominantly characterize the clinical expression of disease and do not simultaneously integrate right ventricular adaptation to pulmonary arterial load, invasively assessed systemic oxygen balance, and nutritional–inflammatory and immunological reserve.

Right ventricular adaptation to pulmonary vascular load is a principal determinant of symptoms, disease progression, and survival in PAH. The ratio of tricuspid annular plane systolic excursion to systolic pulmonary artery pressure (TAPSE/sPAP) provides a readily obtainable, noninvasive surrogate of right ventricular–pulmonary arterial coupling. Lower TAPSE/sPAP values indicate impaired right ventricular systolic adaptation relative to afterload and have been associated with increased mortality and clinical worsening. TAPSE/sPAP has consequently been incorporated into comprehensive guideline-based risk assessment and may provide prognostic information not captured by simplified noninvasive scores [1,5]. Mixed venous oxygen saturation (SvO_2_), assessed during right heart catheterization, provides complementary physiological information by reflecting the integrated balance between systemic oxygen delivery and tissue utilization. In patients with idiopathic or heritable PAH, SvO_2_ demonstrated stronger prognostic performance than thermodilution-derived cardiac index and remained independently associated with long-term mortality [6].

Nutritional–inflammatory and immunological reserve represents another potentially important but incompletely incorporated prognostic domain. The Prognostic Nutritional Index (PNI), calculated from serum albumin concentration and peripheral lymphocyte count, provides a simple and readily available measure reflecting nutritional, inflammatory, and immunological status. Lower PNI values have been associated with increased mortality in primary pulmonary hypertension and with death or lung transplantation in treatment-naïve patients with PAH [7,8]. More recent evidence has also linked impaired nutritional indices to functional limitation, biomarker elevation, hemodynamic impairment, and adverse outcomes in PAH, although the relative prognostic performance of different nutritional tools remains uncertain [9]. Despite this accumulating evidence, nutritional reserve is not represented in the principal guideline-based or simplified PAH risk-assessment instruments.

We therefore developed the Coupling, Oxygen Saturation, and Nutritional Reserve Evaluation in Pulmonary Hypertension (CORE-PH) score, a three-component instrument integrating TAPSE/sPAP, SvO_2_, and PNI. We evaluated its association with long-term all-cause mortality in a treatment-naïve precapillary pulmonary hypertension cohort composed predominantly of patients with WHO group 1 PAH, with a small subgroup of patients with group 4 chronic thromboembolic pulmonary hypertension. We further assessed survival across CORE-PH risk categories and compared its same-cohort discriminatory performance with REVEAL Lite 2 and COMPERA 2.0. Bootstrap internal validation, calibration analysis, and exploratory decision curve analysis were performed to examine model stability, agreement between predicted and observed risk, and potential net clinical benefit.

## 2. Materials and Methods

### 2.1. Study Design and Population

This retrospective, single-center observational cohort study included consecutive adult patients who underwent right heart catheterization (RHC) for the evaluation of suspected or established pulmonary hypertension at the Department of Cardiology, Bursa Uludağ University Faculty of Medicine, Bursa, Türkiye, from 1 January 2011, through 31 December 2025.

The index evaluation was defined as the first eligible diagnostic RHC performed before the initiation of pulmonary arterial hypertension (PAH)-specific therapy, together with the closest available baseline clinical, laboratory, and echocardiographic assessments. For patients who underwent more than one catheterization during the study period, only the first eligible diagnostic procedure was considered.

Adult patients with a final diagnosis of World Health Organization (WHO) group 1 pulmonary arterial hypertension (PAH) or group 4 chronic thromboembolic pulmonary hypertension (CTEPH) were eligible for the primary cohort. Because the study period extended from 2011 to 2025, diagnostic definitions evolved over time. To assess the robustness of the findings under the contemporary hemodynamic definition, all patients were subsequently reclassified using the original unrounded hemodynamic measurements according to the current definition of precapillary pulmonary hypertension: mean pulmonary artery pressure (mPAP) > 20 mmHg, pulmonary artery wedge pressure (PAWP) ≤ 15 mmHg, and pulmonary vascular resistance (PVR) > 2 Wood units. Patients meeting all three contemporary hemodynamic criteria constituted the contemporary-definition sensitivity cohort. Patients with pulmonary hypertension attributable to left-heart disease, reduced left ventricular ejection fraction, advanced left-sided valvular disease, PAWP ≥ 16 mmHg, lung disease or hypoxia, or other noneligible conditions were excluded. Additional exclusions comprised repeat or non-index catheterizations, RHC performed after the initiation of PAH-specific therapy, absence of hemodynamically confirmed pulmonary hypertension, and incomplete catheterization, echocardiographic, or laboratory data [1].

**Figure 1:**
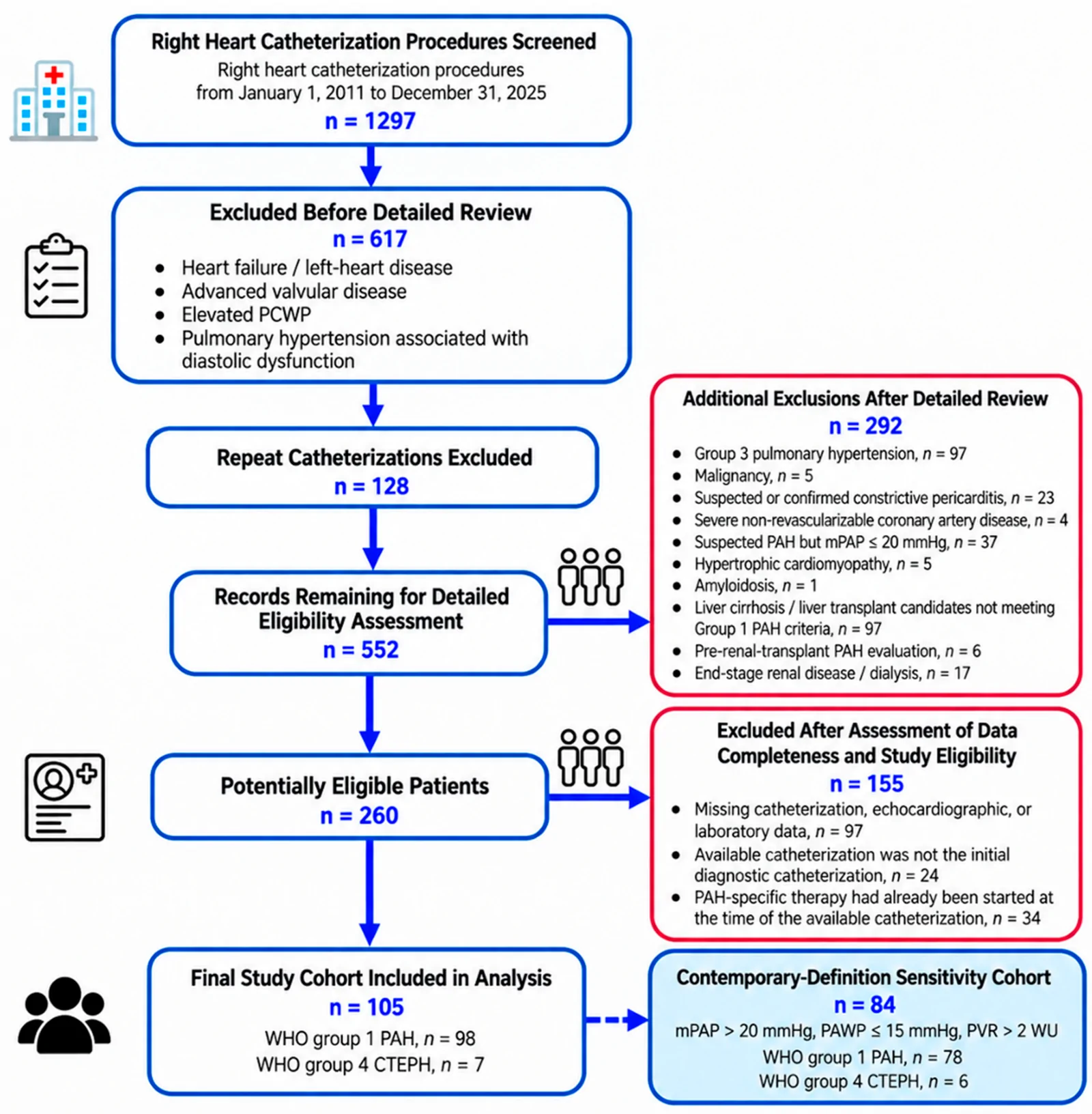
Study flow diagram.

Among 1297 RHC procedures screened, 105 PAH-specific-treatment-naïve patients with WHO group 1 PAH or group 4 CTEPH constituted the final study cohort. The detailed patient-selection process and individual reasons for exclusion are presented in Figure 1.

The study was conducted in accordance with the principles of the Declaration of Helsinki and was approved by the Bursa Uludağ University Health Research Ethics Committee (approval number: 2026/476/15-45; approval date: 26 August 2026). The requirement for written informed consent was waived by the ethics committee because of the retrospective nature of the study.

### 2.2. Data Collection and Clinical Definitions

Demographic characteristics, comorbidities, pulmonary hypertension classification, WHO functional class (WHO-FC), six-minute walk distance (6MWD), history of syncope, laboratory measurements, echocardiographic parameters, invasive hemodynamic findings, and follow-up data were retrospectively obtained from the institutional electronic medical record and the echocardiography and cardiac catheterization reporting systems.

Baseline laboratory measurements were obtained at the time of the index RHC and before the initiation of PAH-specific therapy. Baseline echocardiographic variables were defined as measurements obtained within 30 days of the index RHC and before the initiation of PAH-specific therapy. When more than one eligible echocardiographic examination was available, the study closest to the index catheterization was used.

Laboratory variables included hemoglobin, creatinine, serum albumin, total lymphocyte count, total cholesterol, high-density lipoprotein cholesterol, low-density lipoprotein cholesterol, triglycerides, C-reactive protein, uric acid, and natriuretic peptides. B-type natriuretic peptide and N-terminal pro-B-type natriuretic peptide were analyzed and reported separately because the institutional natriuretic peptide assay changed during the study period.

The Prognostic Nutritional Index was calculated as follows [10]:

PNI = serum albumin (g/L) + 5 × total lymphocyte count (10^9^/L).

### 2.3. Echocardiographic and Hemodynamic Assessment

Baseline echocardiographic variables included left ventricular ejection fraction, tricuspid regurgitation severity, tricuspid annular plane systolic excursion (TAPSE), systolic pulmonary artery pressure (sPAP), interventricular septal flattening resulting in a D-shaped left ventricle, and pericardial effusion.

Right ventricular–pulmonary arterial interaction was estimated using the echocardiographic TAPSE/sPAP ratio, calculated by dividing TAPSE in millimeters by echocardiographically estimated sPAP in millimeters of mercury. TAPSE/sPAP was considered a practical surrogate of right ventricular systolic adaptation relative to pulmonary arterial load [1,5].

Combined right- and left-heart catheterization was performed through femoral venous and arterial access according to the institutional protocol. A complete hemodynamic and oximetry assessment was performed to identify an oxygen saturation step-up and evaluate the presence and anatomical level of intracardiac shunting. Mixed venous oxygen saturation (SvO_2_) was estimated from caval venous samples using the weighted formula SvO_2_ = [3 × superior vena caval oxygen saturation + inferior vena caval oxygen saturation]/4, with both samples obtained before intracardiac admixture [11]. In the two patients with partial anomalous pulmonary venous return involving the superior vena cava or its tributaries, inferior vena caval saturation alone was used as the mixed venous estimate to avoid contamination by oxygenated pulmonary venous blood.

Cardiac output was calculated using the estimated Fick method, assuming a resting oxygen consumption of 125 mL/min/m^2^ of body surface area [12]. This value was applied uniformly as the standardized estimated-Fick approach because directly measured oxygen consumption was not available for retrospective reconstruction. Assumed oxygen consumption may differ substantially from measured values at the individual-patient level and may therefore introduce error into estimated cardiac output. Cardiac index was calculated by indexing cardiac output to body surface area. Pulmonary vascular resistance was calculated as the difference between mean pulmonary artery pressure and PAWP divided by cardiac output and was expressed in Wood units. The complete catheterization, oximetry, and hemodynamic calculation protocol is provided in Supplementary Methods S1.

Hemodynamic variables included right atrial pressure, mean pulmonary artery pressure, PAWP, cardiac index, pulmonary vascular resistance, and SvO_2_. Only measurements obtained during the first eligible diagnostic catheterization before PAH-specific therapy were included in the analyses.

### 2.4. Established Risk-Assessment Tools

REVEAL Lite 2 and COMPERA 2.0 were retrospectively calculated patient-by-patient using their published algorithms and official calculators, based on variables obtained at the index evaluation [2,3]. REVEAL Lite 2 incorporated WHO functional class, systolic blood pressure, heart rate, 6 min walk distance (6MWD), BNP or NT-proBNP, and renal function. The component scores were assigned as follows: WHO-FC I, II, III, and IV were scored −1, 0, +1, and +2, respectively; systolic blood pressure < 110 mmHg and heart rate > 96 beats/min each contributed +1 point; 6MWD ≥ 440 m, 320 ≤ 440 m, 165 ≤ 320 m, and <165 m contributed −2, −1, 0, and +1 point, respectively; BNP < 50 pg/mL or NT-proBNP < 300 pg/mL contributed −2 points, BNP 200 ≤ 800 pg/mL contributed +1 point, and BNP ≥ 800 pg/mL or NT-proBNP ≥ 1100 pg/mL contributed +2 points, with intermediate biomarker values contributing 0 points; and eGFR < 60 mL/min/1.73 m^2^ contributed +1 point. A correction factor of 6 was added to the component sum. REVEAL Lite 2 scores ≤ 5, 6–7, and ≥8 were classified as low, intermediate, and high risk, respectively.

COMPERA 2.0 was calculated patient-by-patient using the official calculator based on WHO functional class, 6MWD, and BNP or NT-proBNP. The published four-strata categories were used: WHO-FC I–II, III, and IV corresponded to component grades 1, 3, and 4, respectively; 6MWD > 440, 320–440, 165–319, and <165 m corresponded to grades 1–4; and BNP < 50, 50–199, 200–800, and >800 pg/mL or NT-proBNP < 300, 300–649, 650–1100, and >1100 pg/mL corresponded to grades 1–4. The overall low-, intermediate-low-, intermediate-high-, or high-risk stratum generated by the official calculator was recorded for each patient.

Heart rate, systolic blood pressure, WHO functional class, and eGFR were available for all 105 patients. Systolic blood pressure for REVEAL Lite 2 was obtained from invasive aortic pressure measured during the index catheterization. eGFR was calculated using the CKD-EPI equation. A natriuretic peptide measurement was available for every patient; BNP was used in 47 patients and NT-proBNP in 58 according to the institutional assay available during the corresponding period. Formal baseline 6MWD measurements were available in 89 patients. In the remaining 16 patients, all of whom were WHO-FC I, no numerical walking distance was imputed; for comparator-score calculation only, the >440 m category was assigned on the basis of contemporaneous clinical documentation of preserved exercise capacity. Consequently, both comparator scores were calculable for all 105 patients, and no missing-variable zero-point rule was required.

### 2.5. Development of the CORE-PH Score

The CORE-PH score—Coupling, Oxygen Saturation, and Nutritional Reserve Evaluation in Pulmonary Hypertension—was developed to integrate three complementary physiological domains: right ventricular adaptation to pulmonary arterial load, systemic oxygen balance, and nutritional–inflammatory and immunological reserve. These domains were represented by TAPSE/sPAP, SvO_2_, and PNI, respectively. The three components were selected on clinical and pathophysiological grounds rather than through an automated variable-selection procedure, and their prognostic associations were subsequently evaluated using Cox proportional hazards regression. Cohort-derived mortality-discrimination thresholds were identified using receiver operating characteristic analysis with maximization of the Youden index. The resulting thresholds were PNI ≤ 47.7, TAPSE/sPAP ≤ 0.4632 mm/mmHg, and SvO_2_ ≤ 68.1%. Because the adjusted effect estimates of the three dichotomized components were of similar magnitude and a simple additive structure was considered preferable for bedside use, one point was assigned for each adverse criterion. Regression coefficients were not used to derive or optimize differential component weights. The total CORE-PH score therefore ranged from 0 to 3 points. Risk strata were defined within the derivation cohort as low risk for scores of 0–1, intermediate risk for a score of 2, and high risk for a score of 3. Scores 0 and 1 were combined because the score-0 subgroup was small and contained few events, while both exact-score groups showed high observed survival through 60 months. The combined 0–1 category was intended as a parsimonious derivation-defined low-risk grouping rather than as evidence of equivalent risk between scores 0 and 1. These categories were therefore derivation-defined rather than predefined. CORE-PH was evaluated both as a continuous 0–3 score and as a categorical risk-stratification tool.

### 2.6. Outcome and Follow-Up

The primary outcome was all-cause mortality. Follow-up time was calculated from the date of the index RHC to the date of death or the final data-lock date. Vital status and dates of death were obtained from official national government records. Patients without a recorded death were censored on the final data-lock date. Mortality records were last updated on 24 July 2026, which served as the final data-lock date. Subsequent treatment after the index RHC was retrospectively reviewed. Because post-baseline management was heterogeneous and treatment timing was nonuniform, subsequent therapies were summarized descriptively and were not entered as baseline covariates in the prognostic models. Management included PAH-targeted pharmacotherapy, defect-directed interventions in congenital heart disease, and CTEPH-specific medical or surgical treatment.

### 2.7. Statistical Analysis

Continuous variables were presented as mean ± standard deviation when approximately normally distributed and as median with interquartile range otherwise. Categorical variables were presented as counts and percentages.

Baseline characteristics were summarized descriptively according to vital status at the final data-lock date (alive/censored at data lock versus deceased during follow-up). Table 1 was presented descriptively without formal cross-sectional hypothesis testing because follow-up duration and censoring differed between patients. Analyses were based on available cases, and missing values were not imputed. The three CORE-PH components and all variables included in the primary multivariable models were complete in all 105 patients.

**Table 1:** Baseline characteristics according to vital status at data lock.

Variable	Alive/Censored at Data Lock (*n* = 54)	Deceased During Follow-Up (*n* = 51)
Clinical characteristics and comorbidities		
Age, years	51.5 (41.0–67.3)	63.0 (56.0–72.0)
Male sex, *n* (%)	12 (22.2)	10 (19.6)
BMI, kg/m^2^	26.77 ± 4.8	27.58 ± 6.8
Heart rate, beats/min	81.9 ± 10.6	83.2 ± 14.9
PH classification, *n* (%)		
Group 1 PAH	49 (90.7)	49 (96.1)
Group 4 CTEPH	5 (9.3)	2 (3.9)
WHO functional class, *n* (%)		
I	20 (37.0)	12 (23.5)
II	21 (38.9)	14 (27.5)
III	13 (24.1)	22 (43.1)
IV	0 (0.0)	3 (5.9)
6 min walk distance, m (*n* = 47/42)	398.0 ± 97.5	271.7 ± 126.0
Syncope at presentation, *n* (%)	5 (9.3)	1 (2.0)
Hypertension, *n* (%)	22 (40.7)	29 (56.9)
Diabetes mellitus, *n* (%)	7 (13.0)	13 (25.5)
Coronary artery disease, *n* (%)	4 (7.4)	6 (11.8)
Atrial fibrillation, *n* (%)	2 (3.7)	7 (13.7)
Laboratory findings		
Hemoglobin, g/dL	13.1 (11.3–14.3)	12.2 (11.0–13.2)
Creatinine, mg/dL	0.72 (0.64–0.94)	0.77 (0.68–0.91)
eGFR, mL/min/1.73 m^2^	60.0 (60.0–91.0)	60.0 (60.0–87.5)
Albumin, g/L	41.1 ± 4.7	37.5 ± 5.3
Total lymphocyte count, ×10^9^/L	2.01 (1.49–2.58)	1.78 (1.16–2.75)
Total cholesterol, mg/dL	176.0 ± 36.9	172.6 ± 47.2
HDL cholesterol, mg/dL	45.5 (39.3–52.0)	42.0 (32.0–51.0)
LDL cholesterol, mg/dL	105.0 ± 32.8	104.0 ± 35.3
Triglycerides, mg/dL	106 (81–146)	118 (88–155)
CRP, mg/L	2.0 (0.6–5.6)	1.8 (0.5–11.1)
Uric acid, mg/dL	5.3 ± 1.7	6.2 ± 2.0
BNP, pg/mL (*n* = 25/22)	112.0 (28.2–216.5)	134.7 (45.0–363.5)
NT-proBNP, pg/mL (*n* = 29/29)	53.6 (26.0–131.5)	146.0 (99.0–438.5)
Prognostic Nutritional Index (PNI)	51.3 ± 6.7	47.2 ± 8.6
Echocardiographic findings		
LVEF, %	60.7 ± 4.2	59.6 ± 4.4
Tricuspid regurgitation severity, *n* (%)		
Mild	19 (35.2)	10 (19.6)
Moderate	21 (38.9)	17 (33.3)
Severe	14 (25.9)	24 (47.1)
TAPSE, mm	20.3 ± 3.6	17.5 ± 4.6
sPAP, mmHg	43.5 (35.0–65.0)	65.0 (50.0–85.0)
TAPSE/sPAP ratio, mm/mmHg	0.483 (0.263–0.595)	0.280 (0.172–0.417)
D-shaped left ventricle, *n* (%)	12 (22.2)	20 (39.2)
Pericardial effusion, *n* (%)	1 (1.9)	6 (11.8)
Right-heart catheterization findings		
Aortic systolic blood pressure, mmHg	136.0 ± 19.3	144.4 ± 27.3
Aortic diastolic blood pressure, mmHg	72.3 ± 10.7	76.1 ± 14.3
RAP, mmHg	7.5 (5.0–10.3)	8.0 (6.0–12.0)
mPAP, mmHg	27.5 (22.5–48.2)	31.3 (23.0–47.0)
PAWP, mmHg	12.0 (10.0–14.0)	12.0 (10.0–13.0)
Cardiac index, L/min/m^2^	2.78 (2.26–3.62)	2.74 (2.24–3.45)
PVR, Wood units	3.27 (1.97–6.86)	3.55 (2.45–8.75)
SvO_2_, %	70.0 (63.3–74.5)	65.0 (56.0–69.4)
Established risk assessment tools		
REVEAL Lite 2 risk category, *n* (%)		
Low risk	39 (72.2)	24 (47.1)
Intermediate risk	11 (20.4)	11 (21.6)
High risk	4 (7.4)	16 (31.4)
COMPERA 2.0 risk strata, *n* (%)		
Low risk	32 (59.3)	16 (31.4)
Intermediate-low risk	15 (27.8)	17 (33.3)
Intermediate-high risk	7 (13.0)	13 (25.5)
High risk	0 (0.0)	5 (9.8)

Data are presented as mean ± standard deviation, median (interquartile range), or *n* (%), as appropriate. Patients without a recorded death were alive and administratively censored at the final data-lock date. This table is therefore presented descriptively, without cross-sectional hypothesis testing. Analyses were based on available cases, and group-specific sample sizes are presented as alive/censored at data lock/deceased during follow-up where applicable. BNP and NT-proBNP were reported separately because the institutional natriuretic peptide assay changed during the study period. Formal associations of baseline variables with all-cause mortality were evaluated using Cox regression and are presented in Table 2. Abbreviations: BMI, body mass index; BNP, B-type natriuretic peptide; CRP, C-reactive protein; CTEPH, chronic thromboembolic pulmonary hypertension; HDL, high-density lipoprotein; LDL, low-density lipoprotein; LVEF, left ventricular ejection fraction; mPAP, mean pulmonary artery pressure; NT-proBNP, N-terminal pro-B-type natriuretic peptide; PAH, pulmonary arterial hypertension; PAWP, pulmonary artery wedge pressure; PH, pulmonary hypertension; PNI, prognostic nutritional index; PVR, pulmonary vascular resistance; RAP, right atrial pressure; sPAP, systolic pulmonary artery pressure; SvO_2_, mixed venous oxygen saturation; TAPSE, tricuspid annular plane systolic excursion; WHO, World Health Organization.

**Table 2:** Univariable and multivariable Cox proportional hazards regression analyses of predictors of all-cause mortality.

Variable	Univariable Analysis				Multivariable Analysis			
	HR	95% CI		*p* Value	HR	95% CI		*p* Value
Age	1.034	1.012	1.057	0.002	1.019	0.995	1.043	0.120
WHO-FC III-IV	2.353	1.353	4.092	0.002	1.689	0.931	3.064	0.085
PNI ≤ 47.7	2.688	1.540	4.692	<0.001	2.681	1.469	4.892	0.001
TAPSE/sPAP ≤ 0.4632	3.866	1.816	8.230	<0.001	2.966	1.344	6.546	0.007
SvO_2_ ≤ 68.1%	2.643	1.428	4.892	0.002	2.404	1.278	4.519	0.006
Male sex	1.023	0.512	2.044	0.948				
BMI	1.023	0.975	1.074	0.349				
Group 1 PAH vs. Group 4 CTEPH	2.148	0.522	8.841	0.290				
WHO-FC				0.010				
Class II vs. I	1.118	0.516	2.420	0.778				
Class III vs. I	2.340	1.152	4.751	0.019				
Class IV vs. I	5.108	1.411	18.493	0.013				
6MWD	0.994	0.992	0.996	<0.001				
Albumin	0.921	0.879	0.965	<0.001				
Uric acid	1.204	1.062	1.365	0.004				
PNI	0.958	0.926	0.991	0.013				
TAPSE	0.894	0.838	0.953	<0.001				
sPAP	1.013	1.004	1.023	0.007				
TAPSE/sPAP	0.061	0.012	0.300	<0.001				
SvO_2_	0.962	0.943	0.982	<0.001				
Severe TR	1.807	1.041	3.134	0.035				

The multivariable model included age, WHO-FC III–IV, PNI ≤ 47.7, TAPSE/sPAP ≤ 0.4632, and SvO_2_ ≤ 68.1%. All variables were entered simultaneously into the model. Hazard ratios for dichotomized variables represent values at or below the specified cut-off compared with values above the cut-off. Abbreviations: CI, confidence interval; HR, hazard ratio; PNI, prognostic nutritional index; sPAP, systolic pulmonary artery pressure; SvO_2_, mixed venous oxygen saturation; TAPSE, tricuspid annular plane systolic excursion; WHO-FC, World Health Organization functional class.

Associations between baseline variables and all-cause mortality were evaluated using Cox proportional hazards regression. Results were reported as hazard ratios (HRs) with 95% confidence intervals (CIs). Univariable Cox regression analyses were initially performed for clinically relevant demographic, functional, laboratory, echocardiographic, and hemodynamic variables.

Given the 51 observed deaths, the main multivariable Cox model was restricted to five clinically relevant variables to maintain model parsimony. The model included age, WHO-FC III–IV, PNI ≤ 47.7, TAPSE/sPAP ≤ 0.4632, and SvO_2_ ≤ 68.1%. All variables were entered simultaneously. For dichotomized variables, HRs represented values at or below the specified threshold compared with values above that threshold.

The prognostic association of CORE-PH was subsequently evaluated in separate Cox models. First, CORE-PH was entered as a continuous score to estimate the mortality hazard associated with each one-point increase. Second, CORE-PH was entered as a categorical variable, with the low-risk group serving as the reference category. Both models were adjusted for age and WHO-FC III–IV.

The proportional hazards assumption was evaluated using scaled Schoenfeld residuals and global proportionality tests. Because age demonstrated evidence of a time-varying effect, sensitivity analyses were performed using centered age and an age-by-log(time + 0.1) interaction term. The associations of the continuous CORE-PH score and categorical CORE-PH risk groups with mortality were reassessed in these time-varying coefficient models. The primary multivariable component model was also reassessed using the same time-varying age specification, with centered age and an age-by-log(time + 0.1) interaction term while retaining WHO-FC III–IV and the three dichotomized CORE-PH components. To assess potential calendar-era effects across the 2011–2025 inclusion period, an additional sensitivity model included index calendar year as a continuous covariate together with centered age, WHO-FC III–IV, the continuous CORE-PH score, and the age-by-log(time + 0.1) interaction term.

To assess robustness to the contemporary hemodynamic definition, Cox regression, Kaplan–Meier survival analysis, and time-dependent discrimination analyses were repeated in patients meeting mPAP > 20 mmHg, PAWP ≤ 15 mmHg, and PVR > 2 Wood units at the index catheterization. This contemporary-definition sensitivity cohort comprised 84 patients. To further assess the potential influence of estimated-Fick cardiac output on PVR-based classification, an additional stringent sensitivity analysis was performed after restricting the contemporary-definition cohort to patients with PVR > 3 Wood units at the index catheterization. Because COMPERA 2.0 and REVEAL Lite 2 were developed for patients with PAH, additional sensitivity analyses were performed in the primary cohort after exclusion of the seven patients with CTEPH. In this primary-cohort PAH-only sensitivity analysis, the adjusted association of CORE-PH with mortality, categorical risk-group associations, and time-dependent discrimination relative to COMPERA 2.0 and REVEAL Lite 2 were reassessed using the same analytical framework as in the primary cohort. The original CORE-PH thresholds and risk-group definitions were retained in all sensitivity analyses.

Kaplan–Meier survival curves were generated according to CORE-PH risk category and compared using the log-rank test. Median survival times and corresponding 95% CIs were estimated where calculable.

Discrimination of CORE-PH, COMPERA 2.0, and REVEAL Lite 2 was evaluated using time-dependent receiver operating characteristic analysis at 12, 36, and 60 months to account for censoring and differences in follow-up duration. Time-dependent areas under the curve (AUCs) and corresponding 95% confidence intervals were estimated using marginal inverse-probability-of-censoring weighting. Because all three risk scores were evaluated in the same patients, paired comparisons of time-dependent AUCs were performed between CORE-PH and each comparator at each time horizon. Conventional binary ROC analysis with maximization of the Youden index was used only for derivation of the CORE-PH component thresholds and was not used for the final comparative assessment of prognostic discrimination [13].

Internal validation of the Cox model containing the continuous 0–3 CORE-PH score was performed using 1000 bootstrap resamples [14]. In each resample, the three component thresholds were re-estimated using the same Youden-index procedure, the binary components were reconstructed, the equal one-point weighting rule was reapplied to generate the 0–3 CORE-PH score, and the Cox score model was refitted. Model performance was evaluated in both the bootstrap sample and the original cohort. Optimism was estimated as the mean difference between bootstrap-sample and test-sample performance and was subtracted from the apparent Harrell C-index. An optimism-corrected calibration slope was calculated using the same bootstrap framework.

Apparent calibration of predicted 36-month mortality was assessed across the four exact CORE-PH scores. Model-predicted 36-month mortality probabilities were derived from the Cox model fitted with the continuous 0–3 CORE-PH score in the original cohort and evaluated at scores of 0, 1, 2, and 3. For each score, observed 36-month mortality was estimated using the Kaplan–Meier method and plotted against the corresponding model-predicted mortality probability. Ninety-five percent confidence intervals for observed mortality were displayed where estimable.

Exploratory time-dependent decision curve analysis was performed at 12, 36, and 60 months to evaluate the net clinical benefit of CORE-PH across a range of threshold probabilities. The net benefit of CORE-PH was compared with that of COMPERA 2.0 and REVEAL Lite 2 and with the default strategies of treating all patients and treating no patients [15].

All statistical tests were two-sided, and a *p*-value < 0.05 was considered statistically significant. Statistical analyses were performed using IBM SPSS Statistics, version 29.0 (IBM Corp., Armonk, NY, USA), and R software, version 4.6.1 (R Foundation for Statistical Computing, Vienna, Austria). Reporting of prediction model development and internal validation was guided by the TRIPOD+AI statement [16].

## 3. Results

### 3.1. Study Population and Baseline Characteristics

A total of 105 patients met the eligibility criteria and were included in the final analysis (Figure 1). Of these, 98 (93.3%) had World Health Organization group 1 pulmonary arterial hypertension and 7 (6.7%) had group 4 chronic thromboembolic pulmonary hypertension. Among the 98 patients with group 1 PAH, 49 (50.0%) had connective tissue disease-associated PAH, 31 (31.6%) had congenital heart disease-associated PAH, and 18 (18.4%) had other group 1 PAH etiologies. During a median observed follow-up of 70.0 months (interquartile range, 43.8–111.3 months), 51 patients (48.6%) died, whereas 54 (51.4%) were alive and administratively censored at the final data-lock date.

Descriptively, patients who died during follow-up were older, had a less favorable World Health Organization functional class distribution, and had a shorter six-minute walk distance than those who were alive at data lock. Other clinical characteristics and comorbidities are summarized descriptively in Table 1.

Patients who died during follow-up had lower serum albumin concentrations (37.5 ± 5.3 vs. 41.1 ± 4.7 g/L), higher uric acid levels (6.2 ± 2.0 vs. 5.3 ± 1.7 mg/dL), and lower Prognostic Nutritional Index values (47.2 ± 8.6 vs. 51.3 ± 6.7). N-terminal pro-B-type natriuretic peptide concentrations were also higher among patients who died during follow-up [146.0 (99.0–438.5) vs. 53.6 (26.0–131.5) pg/mL], whereas hemoglobin, creatinine, lipid parameters, C-reactive protein, and total lymphocyte count were broadly similar between the groups. Echocardiographic assessment demonstrated lower tricuspid annular plane systolic excursion (17.5 ± 4.6 vs. 20.3 ± 3.6 mm), higher systolic pulmonary artery pressure [65.0 (50.0–85.0) vs. 43.5 (35.0–65.0) mmHg], and lower TAPSE/sPAP ratios [0.280 (0.172–0.417) vs. 0.483 (0.263–0.595) mm/mmHg] among patients who died during follow-up. Right atrial pressure, mean pulmonary artery pressure, pulmonary artery wedge pressure, cardiac index, and pulmonary vascular resistance were broadly similar between the groups. Mixed venous oxygen saturation was lower among patients who died during follow-up [65.0% (56.0–69.4%) vs. 70.0% (63.3–74.5%)].

Established risk-category distributions are summarized descriptively according to vital status at data lock. High-risk REVEAL Lite 2 classification was more frequent among patients who died during follow-up (31.4% vs. 7.4%), and intermediate-high or high COMPERA 2.0 strata were likewise more frequent in this group.

Subsequent management was heterogeneous and included PAH-targeted pharmacotherapy, vasoreactivity-guided calcium-channel blocker therapy in selected patients, defect-directed interventions in congenital heart disease, and CTEPH-specific medical or surgical treatment. Among patients with complete coding for the prespecified PAH-targeted drug-class variables, 53 had at least one such drug class recorded during follow-up. These drug-class variables did not capture all disease-directed treatment modalities.

### 3.2. Predictors of All-Cause Mortality

In univariable Cox regression analyses, older age, advanced World Health Organization functional class, shorter six-minute walk distance, lower albumin, higher uric acid, lower Prognostic Nutritional Index, lower TAPSE, higher sPAP, lower TAPSE/sPAP ratio, lower mixed venous oxygen saturation, and severe tricuspid regurgitation were associated with an increased risk of all-cause mortality (Table 2).

In the main multivariable model, a Prognostic Nutritional Index ≤ 47.7 was independently associated with mortality (hazard ratio [HR], 2.681; 95% confidence interval [CI], 1.469–4.892; *p* = 0.001). A TAPSE/sPAP ratio ≤ 0.4632 was associated with an approximately threefold higher mortality hazard (HR, 2.966; 95% CI, 1.344–6.546; *p* = 0.007), whereas mixed venous oxygen saturation ≤68.1% was associated with a 2.404-fold higher hazard (95% CI, 1.278–4.519; *p* = 0.006). Age and World Health Organization functional class III–IV did not retain statistical significance after simultaneous adjustment. Because age demonstrated evidence of nonproportional hazards, the primary multivariable component model was additionally refitted using a time-varying age term. In this model, all three CORE-PH components remained independently associated with all-cause mortality: PNI ≤ 47.7 (HR, 2.77; 95% CI, 1.52–5.05; *p* < 0.001), TAPSE/sPAP ≤ 0.4632 (HR, 2.92; 95% CI, 1.32–6.45; *p* = 0.008), and SvO_2_ ≤ 68.1% (HR, 2.58; 95% CI, 1.37–4.89; *p* = 0.004). The age-by-log(time + 0.1) interaction was significant (HR, 1.018; 95% CI, 1.005–1.032; *p* = 0.007), and the model C-index was 0.779.

### 3.3. CORE-PH Score Distribution and Association with Mortality

The CORE-PH score ranged from 0 to 3 points. Thirteen patients had a score of 0, 34 had a score of 1, 40 had a score of 2, and 18 had a score of 3. During follow-up, one death occurred among the 13 patients with a score of 0 and seven deaths among the 34 patients with a score of 1. Using the derivation-defined categories, 47 patients were classified as low risk (0–1 points), 40 as intermediate risk (2 points), and 18 as high risk (3 points). Death occurred in 8 of 47 low-risk patients (17.0%), 27 of 40 intermediate-risk patients (67.5%), and 16 of 18 high-risk patients (88.9%).

When analyzed as a continuous variable, each 1-point increase in CORE-PH was associated with a 2.865-fold higher mortality hazard in univariable analysis (95% CI, 2.005–4.096; *p* < 0.001). This association remained robust after adjustment for age and World Health Organization functional class III–IV (adjusted HR, 2.645; 95% CI, 1.823–3.837; *p* < 0.001).

When CORE-PH was analyzed categorically, the overall association between risk category and mortality was significant in both univariable and adjusted analyses (*p* < 0.001 for both). Compared with the low-risk group, intermediate-risk patients had an unadjusted HR of 5.295 (95% CI, 2.403–11.666; *p* < 0.001) and an adjusted HR of 4.359 (95% CI, 1.937–9.811; *p* < 0.001). High-risk patients had an unadjusted HR of 11.000 (95% CI, 4.665–25.939; *p* < 0.001) and an adjusted HR of 9.233 (95% CI, 3.849–22.144; *p* < 0.001; Table 3).

Schoenfeld residual testing demonstrated no evidence of nonproportionality for either the continuous CORE-PH score or its categorical risk groups. A time-varying effect was observed for age. In a sensitivity analysis incorporating an age-by-log(time + 0.1) interaction, the continuous CORE-PH score remained independently associated with mortality (HR per 1-point increase, 2.735; 95% CI, 1.885–3.968; *p* < 0.001). Similarly, the adjusted mortality hazards remained elevated in both the intermediate-risk (HR, 4.469; 95% CI, 1.990–10.038; *p* < 0.001) and high-risk groups (HR, 9.895; 95% CI, 4.137–23.670; *p* < 0.001), confirming the robustness of the primary findings.

After exclusion of the seven patients with CTEPH, the PAH-only sensitivity cohort comprised 98 patients, with 49 deaths during follow-up. CORE-PH remained independently associated with all-cause mortality after adjustment for age and WHO functional class III–IV (HR per 1-point increase, 2.46; 95% CI, 1.71–3.55; *p* < 0.001). Compared with the low-risk group, adjusted mortality hazards were 4.40-fold higher in the intermediate-risk group (95% CI, 1.95–9.92; *p* < 0.001) and 8.06-fold higher in the high-risk group (95% CI, 3.32–19.57; *p* < 0.001).

When the primary cohort was uniformly reclassified using contemporary hemodynamic criteria, 84 of 105 patients met all three requirements for precapillary pulmonary hypertension (mPAP > 20 mmHg, PAWP ≤ 15 mmHg, and PVR > 2 Wood units). The remaining 21 patients were retained in the historical primary cohort but were excluded from this contemporary-definition sensitivity analysis. This contemporary-definition sensitivity cohort included 78 patients with group 1 PAH and 6 with group 4 CTEPH, with 44 deaths occurring during follow-up. CORE-PH remained independently associated with all-cause mortality after adjustment for age and WHO functional class III–IV (HR per 1-point increase, 2.48; 95% CI, 1.57–3.93; *p* < 0.001). Compared with the low-risk group, adjusted mortality hazards were 3.46-fold higher in the intermediate-risk group (95% CI, 1.27–9.45; *p* = 0.016) and 7.29-fold higher in the high-risk group (95% CI, 2.51–21.18; *p* < 0.001). In a further stringent sensitivity analysis restricted to patients with PVR > 3 Wood units (*n* = 60; 31 deaths), CORE-PH remained independently associated with all-cause mortality after adjustment for age and WHO-FC III–IV (HR per 1-point increase, 2.71; 95% CI, 1.43–5.12; *p* = 0.002; C-index, 0.754).

Separately, in the primary cohort, an additional sensitivity analysis accounting for index calendar year and the time-varying effect of age showed that CORE-PH remained independently associated with all-cause mortality (HR per 1-point increase, 3.01; 95% CI, 2.08–4.36; *p* < 0.001; C-index, 0.787).

### 3.4. Time-Dependent Discrimination Compared with Established Risk Tools

**Figure 2:**
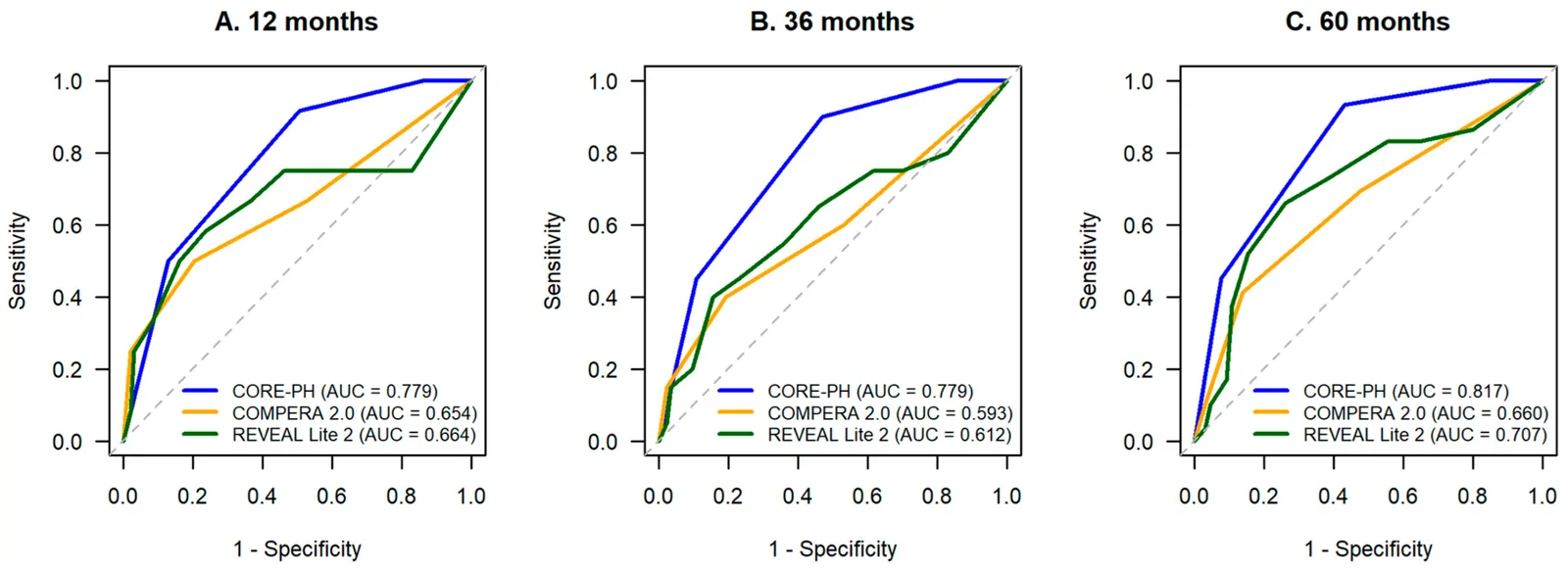
Time-dependent receiver operating characteristic curves comparing CORE-PH, COMPERA 2.0, and REVEAL Lite 2 for discrimination of all-cause mortality at 12 months (**A**), 36 months (**B**), and 60 months (**C**) in the primary cohort. Time-dependent AUCs were estimated using marginal inverse-probability-of-censoring weighting. The dashed diagonal line represents chance-level discrimination (AUC = 0.50).

Time-dependent discrimination was evaluated at 12, 36, and 60 months. CORE-PH yielded AUCs of 0.779 (95% CI, 0.663–0.894), 0.779 (95% CI, 0.683–0.875), and 0.817 (95% CI, 0.738–0.896), respectively. The corresponding AUCs for COMPERA 2.0 were 0.654 (95% CI, 0.470–0.838), 0.593 (95% CI, 0.448–0.738), and 0.660 (95% CI, 0.543–0.777), whereas those for REVEAL Lite 2 were 0.664 (95% CI, 0.460–0.869), 0.612 (95% CI, 0.460–0.765), and 0.707 (95% CI, 0.587–0.827). Pairwise comparisons showed no significant difference between CORE-PH and COMPERA 2.0 at 12 months (*p* = 0.279), whereas CORE-PH demonstrated higher time-dependent AUCs at 36 months (*p* = 0.032) and 60 months (*p* = 0.021). Differences between CORE-PH and REVEAL Lite 2 were not statistically significant at 12 months (*p* = 0.350), 36 months (*p* = 0.057), or 60 months (*p* = 0.106; Table 4 and Figure 2).

In the PAH-only sensitivity cohort (*n* = 98), time-dependent AUCs for CORE-PH were 0.761 (95% CI, 0.639–0.882), 0.768 (95% CI, 0.668–0.867), and 0.814 (95% CI, 0.732–0.895) at 12, 36, and 60 months, respectively. The corresponding AUCs were 0.640 (95% CI, 0.447–0.832), 0.586 (95% CI, 0.439–0.734), and 0.665 (95% CI, 0.546–0.783) for COMPERA 2.0 and 0.654 (95% CI, 0.435–0.872), 0.611 (95% CI, 0.453–0.768), and 0.719 (95% CI, 0.596–0.841) for REVEAL Lite 2. CORE-PH showed higher discrimination than COMPERA 2.0 at 36 months (*p* = 0.045) and 60 months (*p* = 0.035), but not at 12 months (*p* = 0.329). Differences versus REVEAL Lite 2 remained non-significant at all three horizons (*p* = 0.420, *p* = 0.086, and *p* = 0.179, respectively).

In the contemporary-definition sensitivity cohort (*n* = 84), time-dependent AUCs for CORE-PH were 0.732, 0.732, and 0.778 at 12, 36, and 60 months, respectively. Pairwise differences versus COMPERA 2.0 and REVEAL Lite 2 were not statistically significant at any evaluated horizon.

### 3.5. Survival According to CORE-PH Risk Category

Before aggregation into risk categories, outcomes for exact CORE-PH scores of 0 and 1 were reported separately. Among patients with a score of 0 (*n* = 13), one death occurred during the entire follow-up, with no deaths within the first 60 months; Kaplan–Meier survival was 100% at 12, 36, and 60 months, and median survival was not reached, with its 95% confidence interval not estimable. Among patients with a score of 1 (*n* = 34), seven deaths occurred during follow-up; Kaplan–Meier survival was 97.1% at 12 months and 94.1% at both 36 and 60 months, and median survival was likewise not reached, with its 95% confidence interval not estimable. Given the persistently high observed survival through 60 months in both exact-score groups and the small size and sparse event count of the score-0 subgroup, scores 0 and 1 were combined into the derivation-defined low-risk category.

**Figure 3:**
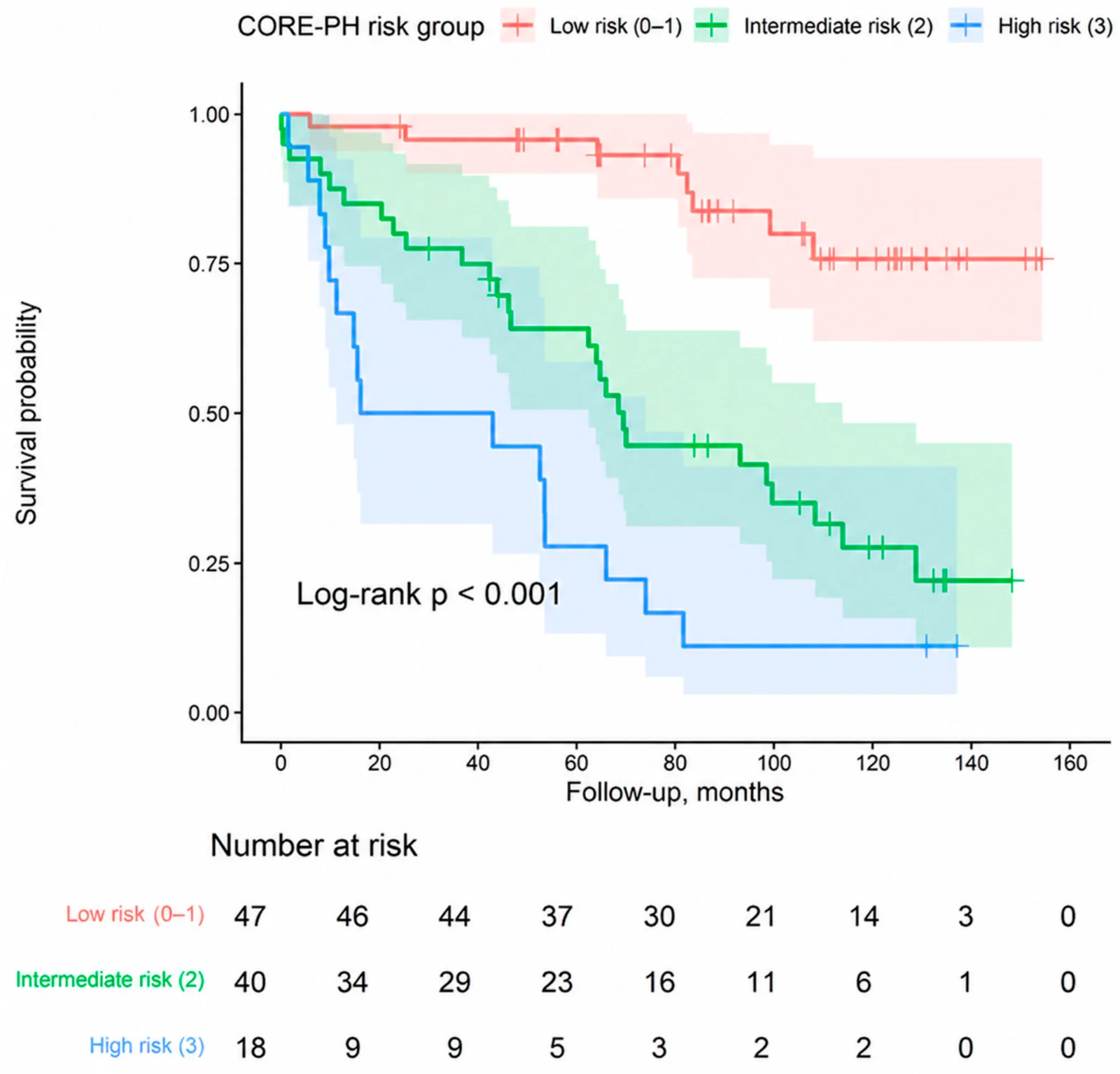
Kaplan–Meier survival curves according to CORE-PH risk categories. Survival decreased progressively across the low-risk (0–1 points), intermediate-risk (2 points), and high-risk (3 points) groups. Shaded areas represent 95% confidence intervals for the Kaplan–Meier survival estimates. Median survival was not reached in the low-risk group, and its 95% confidence interval was not estimable; median survival was 69.5 months (95% CI, 62.4–113.9) in the intermediate-risk group and 29.5 months (95% CI, 11.2–74.0) in the high-risk group (log-rank χ^2^ = 40.780; *p* < 0.001).

Kaplan–Meier analysis demonstrated a marked and progressive separation of survival curves across the three CORE-PH risk categories (log-rank χ^2^ = 40.780; *p* < 0.001; Figure 3). Median survival was not reached in the low-risk group, and its 95% confidence interval was not estimable. Median survival was 69.5 months (95% CI, 62.4–113.9) in the intermediate-risk group and 29.5 months (95% CI, 11.2–74.0) in the high-risk group.

### 3.6. Internal Validation and Calibration

Bootstrap internal validation incorporating repeated threshold optimization and score reconstruction was performed using 1000 resamples, all of which were successfully completed. The apparent Harrell C-index was 0.747. Mean estimated optimism was 0.028, yielding an optimism-corrected C-index of 0.719. The apparent calibration slope was 1.000, and the optimism-corrected calibration slope was 0.888 (Table 5). These findings indicate a measurable reduction in model performance after accounting for same-cohort derivation and threshold optimization.

**Figure 4:**
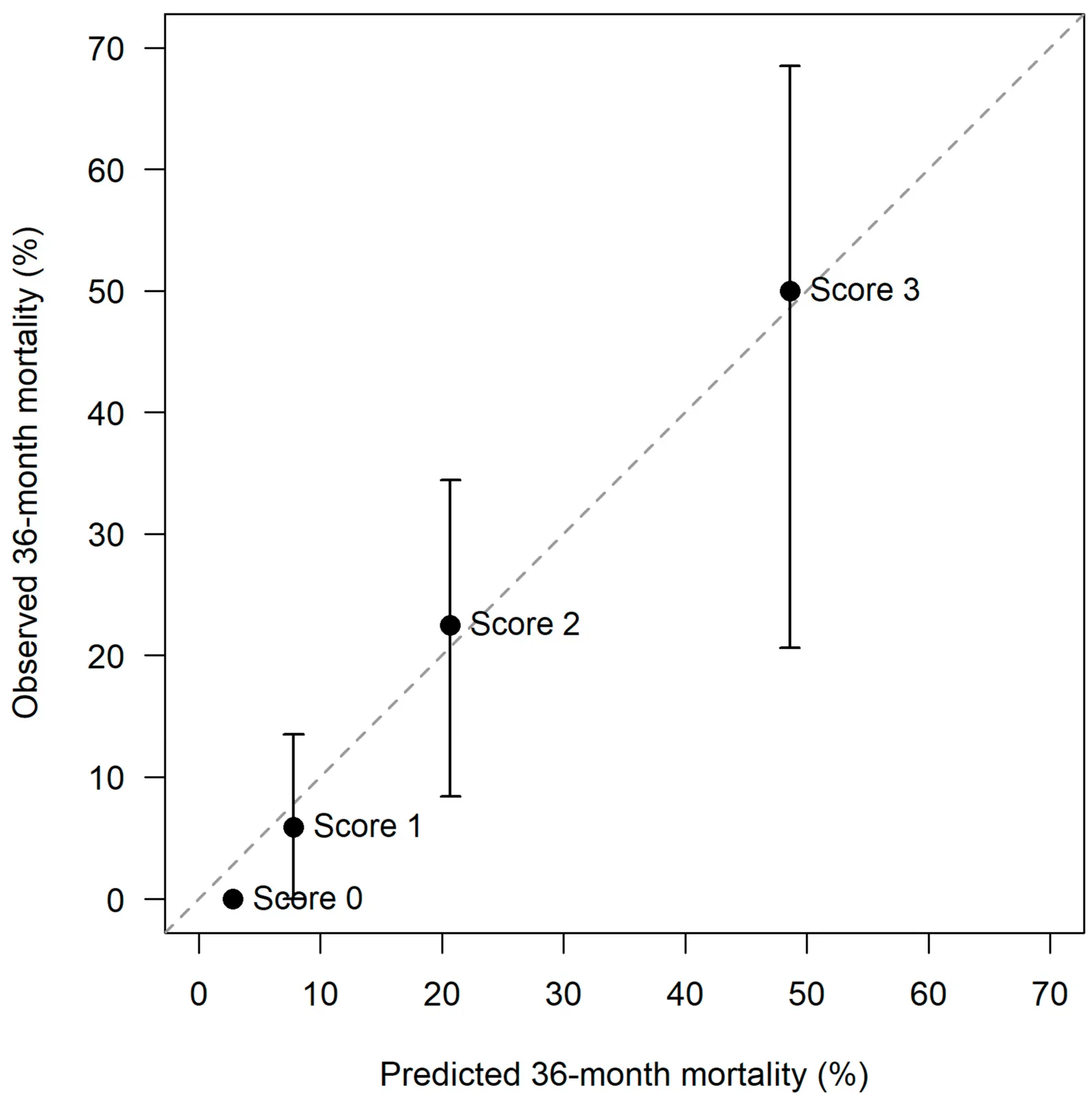
Apparent calibration of CORE-PH for 36-month mortality according to exact score. Observed 36-month mortality probabilities were estimated using the Kaplan–Meier method and plotted against model-predicted mortality probabilities for CORE-PH scores 0–3. The dashed diagonal line represents perfect calibration. Error bars indicate 95% confidence intervals where estimable.

At 36 months, apparent calibration across the four exact CORE-PH scores showed close agreement between model-predicted and observed mortality probabilities (Figure 4). Observed mortality was 0.0% for score 0, 5.9% (95% CI, 0–13.5%) for score 1, 22.5% (95% CI, 8.4–34.4%) for score 2, and 50.0% (95% CI, 20.6–68.5%) for score 3, compared with corresponding model-predicted probabilities of 2.8%, 7.8%, 20.7%, and 48.6%, respectively.

### 3.7. Decision Curve Analysis

In exploratory decision curve analyses, CORE-PH provided greater apparent net benefit than COMPERA 2.0 and REVEAL Lite 2 across most low-to-moderate threshold probabilities. The relative clinical benefit of CORE-PH was most consistent at the 36- and 60-month time horizons, whereas separation between the models was less pronounced at 12 months. These findings were also favorable relative to the default treat-all and treat-none strategies (Supplementary Figure S1).

## 4. Discussion

In this treatment-naïve cohort of patients with predominantly group 1 pulmonary arterial hypertension, the newly developed CORE-PH score provided a simple, physiologically grounded framework for long-term mortality stratification. Each one-point increase in CORE-PH was associated with an approximately 2.6-fold higher adjusted mortality hazard, while intermediate- and high-risk categories showed progressively greater risks compared with the low-risk category. Using censoring-aware time-dependent analyses, CORE-PH yielded AUCs of 0.779, 0.779, and 0.817 at 12, 36, and 60 months, respectively. CORE-PH demonstrated higher discrimination than COMPERA 2.0 at 36 and 60 months, whereas differences versus REVEAL Lite 2 were not statistically significant at any evaluated time horizon. Bootstrap internal validation incorporating repeated threshold optimization and score reconstruction yielded an optimism-corrected C-index of 0.719 and an optimism-corrected calibration slope of 0.888, indicating measurable optimism after accounting for same-cohort development. Collectively, these findings support further evaluation of the combined assessment of right ventricular–pulmonary arterial adaptation, systemic oxygen balance, and nutritional–immunological reserve.

Contemporary PAH management regards risk assessment as a repeated, multidimensional process rather than a single prognostic calculation. The 2022 ESC/ERS guidelines recommend a comprehensive three-strata assessment at diagnosis, incorporating as many clinically relevant domains as possible, and a simplified four-strata approach during follow-up, with achievement or maintenance of low-risk status as the principal treatment objective [1]. More recently, the ISHLT consensus statement emphasized risk assessment at baseline and at regular follow-up, its integration with clinical judgement, and the importance of capturing the full spectrum of risk relevant to treatment escalation and advanced-care planning [17]. Within this framework, REVEAL Lite 2 and COMPERA 2.0 are appropriate benchmark comparators. REVEAL Lite 2 is a weighted six-variable noninvasive score developed to discriminate 1-year mortality risk, whereas COMPERA 2.0 uses WHO functional class, 6 min walk distance, and BNP or NT-proBNP to divide the heterogeneous intermediate-risk population into intermediate-low and intermediate-high strata [2,3]. Although developed for partly different clinical settings and time horizons, both have subsequently demonstrated associations with longer-term outcomes and remain clinically relevant reference standards [4,17].

CORE-PH was not designed as a reduced version of either established tool. Instead, it was constructed around a distinct conceptual premise: prognosis in precapillary pulmonary hypertension may depend not only on the magnitude of pulmonary vascular disease, but also on the progressive exhaustion of the physiological reserves that permit the patient to tolerate that disease. TAPSE/sPAP represents the adequacy of the mechanical response of the right ventricle to pulmonary arterial load; SvO_2_ reflects whether the resulting circulation remains sufficient to preserve systemic oxygen delivery–extraction balance; and PNI reflects the broader nutritional, inflammatory, and immunological reserve of the organism. These components may therefore be viewed as successive but nonredundant levels within a pathophysiological continuum extending from impaired right ventricular adaptation to inadequate systemic oxygen balance and, ultimately, systemic vulnerability.

The prognostic contribution of TAPSE/sPAP is consistent with the central role of right ventricular adaptation in PAH. Pulmonary vascular obstruction increases effective arterial load, to which the right ventricle initially responds by increasing contractility and wall thickness. This adaptive response may preserve stroke volume and ventriculoarterial coupling despite markedly increased pulmonary pressures. With disease progression, however, further augmentation of contractility becomes unsustainable, right ventricular dilatation and wall stress increase, and mechanical efficiency deteriorates [17]. TAPSE/sPAP provides a readily available echocardiographic surrogate of the adequacy of longitudinal right ventricular systolic performance relative to the pressure load imposed by the pulmonary circulation. It should not be regarded as a direct measurement of pressure–volume loop-derived Ees/Ea, but it has repeatedly demonstrated prognostic value in PAH [5,18,19].

The TAPSE/sPAP threshold identified in the present study, 0.4632 mm/mmHg, was higher than the thresholds used in the ESC/ERS three-strata model, in which values above 0.32, between 0.19 and 0.32, and below 0.19 mm/mmHg correspond to low-, intermediate-, and high-risk profiles, respectively [1]. Accordingly, the present threshold should not be interpreted as a universal boundary for established RV–PA uncoupling. It may instead identify a less advanced but prognostically relevant reduction in right ventricular adaptation or erosion of coupling reserve. This interpretation is supported by the dynamic nature of RV adaptation and by recent evidence showing that lower baseline TAPSE/sPAP is associated with mortality and clinical worsening across PAH cohorts. A recent meta-analysis of 14 studies involving 3288 patients confirmed a consistent inverse association between TAPSE/sPAP and adverse outcomes, while also showing that TAPSE/sPAP does not uniformly correspond to invasively measured Ees/Ea [19]. The 2025 ISHLT consensus similarly recognizes that incorporating right ventricular imaging variables into established risk tools may improve discrimination, particularly in patients otherwise classified within low- or intermediate-risk ranges [17,20]. Cardiac magnetic resonance (CMR) data were not available in the present cohort and therefore could not be incorporated into the CORE-PH framework. CMR provides robust quantitative assessment of right ventricular function and remodeling, and meta-analytic evidence in PAH has demonstrated the prognostic value of right ventricular ejection fraction and right ventricular end-diastolic and end-systolic volumes for mortality and clinical worsening [21,22]. Future studies should therefore evaluate whether CMR-derived RV functional and volumetric measures provide incremental prognostic information beyond the echocardiographic TAPSE/sPAP domain incorporated in CORE-PH.

SvO_2_ provided a complementary physiological domain. Cardiac output quantifies forward flow but does not establish whether that flow is sufficient for the prevailing systemic oxygen requirement. SvO_2_ integrates cardiac output with arterial oxygen content, hemoglobin availability, and tissue oxygen extraction, and therefore reflects the residual oxygen content of venous blood after systemic utilization. Two patients with a similar cardiac index may have substantially different SvO_2_ values because of differences in hemoglobin concentration, arterial oxygenation, metabolic demand, or extraction. This distinction may help explain why SvO_2_ remained prognostically informative despite broadly similar cardiac index values across vital-status groups.

Khirfan et al. demonstrated only moderate correlation between thermodilution cardiac index and SvO_2_ and concordant guideline risk classification in fewer than half of patients with idiopathic or heritable PAH. SvO_2_-based risk categories separated long-term survival, whereas cardiac index categories did not, and SvO_2_ remained associated with mortality after adjustment for age and sex [6]. Nagata et al. subsequently showed that reduced mixed venous oxygen tension identified a tissue-hypoxia phenotype associated with poor long-term outcomes in treatment-naïve PAH and medically treated CTEPH, further supporting the prognostic relevance of mixed venous oxygenation beyond flow measurements alone [23]. Thus, within CORE-PH, SvO_2_ may be regarded as the functional consequence of right ventricular adaptation: it indicates whether the circulation generated by the right ventricle remains adequate relative to systemic metabolic requirements.

The cohort-derived SvO_2_ threshold of 68.1% was higher than the conventional ESC/ERS thresholds of >65%, 60–65%, and <60% used to define low-, intermediate-, and high-risk hemodynamic profiles [1]. Values below 60% generally indicate advanced impairment of circulatory reserve and increased systemic extraction. By contrast, the present threshold may reflect an earlier or less severe reduction in oxygen-balance reserve associated with long-term mortality before overt low-output physiology develops. This remains a physiological interpretation rather than a demonstrated temporal sequence and should be tested in serial studies. The threshold may also be influenced by the baseline, PAH-specific-treatment-naïve setting and by the longer outcome horizon of the present analysis.

Several design features strengthen the interpretation of SvO_2_ in this cohort. Patients with reduced left ventricular ejection fraction, advanced left-sided valvular disease, post-capillary pulmonary hypertension, or PAWP ≥16 mmHg were excluded, reducing the likelihood that low SvO_2_ primarily reflected left-heart-mediated low-output physiology or pulmonary venous congestion. Furthermore, the institutional catheterization protocol included a systematic saturation run from the inferior and superior vena cava, multiple right atrial levels, right ventricle, pulmonary artery, left ventricle, and aorta. Mixed venous saturation was derived from caval samples obtained before intracardiac admixture; when anomalous pulmonary venous return affected the superior caval territory, inferior vena caval saturation was used to avoid contamination by oxygenated pulmonary venous blood. This approach limits—but cannot completely eliminate—the influence of shunt physiology and sampling variability on systemic venous oxygenation.

The third component, PNI, extends the model beyond cardiovascular mechanics and invasive hemodynamics. Malnutrition in PAH is unlikely to represent inadequate intake alone. Right-sided congestion may contribute to bowel-wall edema, impaired absorption, hepatic dysfunction, early satiety, and reduced appetite, while chronic inflammation, increased respiratory work, neurohormonal activation, and catabolic stress may further impair nutritional status. PNI combines serum albumin with peripheral lymphocyte count and therefore reflects more than nutritional intake; it provides a pragmatic estimate of nutritional–inflammatory and immunological reserve. Nevertheless, PNI should not be considered a formal diagnostic criterion for malnutrition, which requires broader phenotypic and etiological assessment.

Previous studies support the prognostic relevance of this domain. In a historical primary pulmonary hypertension registry, lower PNI was associated with greater all-cause mortality even after adjustment for functional and hemodynamic variables [7]. In a contemporary treatment-naïve PAH cohort, Nakashima et al. showed that PNI, GNRI, and CONUT were independently associated with death or lung transplantation over a median follow-up of 5.5 years [8]. Subsequent work has linked impaired nutritional indices to functional limitation, neurohormonal activation, hemodynamic severity, and adverse outcomes [9]. The PNI threshold of 47.7 identified in the present study is biologically compatible with the distributions reported in previous PAH cohorts, but it remains cohort-derived and should not be interpreted as a universal malnutrition threshold. Its value within CORE-PH lies in representing a prognostic domain that is not directly incorporated into REVEAL Lite 2 or COMPERA 2.0.

The comparison with established risk tools requires careful interpretation. REVEAL Lite 2 and COMPERA 2.0 are not merely mortality equations; they are dynamic clinical instruments designed to identify residual risk, evaluate treatment response, and support therapeutic escalation toward a low-risk state. In PATENT, both baseline REVEAL Lite 2 and early change in score were associated with survival and clinical worsening-free survival [24]. In GRIPHON, improvement in REVEAL Lite 2 was associated with reduced morbidity/mortality risk and accounted for a proportion of the treatment effect of selexipag, particularly among higher-risk patients [25]. In REPLACE, COMPERA 2.0 was sensitive to changes after switching from a PDE5 inhibitor to riociguat and identified the intermediate-high-risk patients in whom clinical worsening occurred [26]. CORE-PH has not yet been evaluated for these longitudinal or treatment-monitoring functions.

The present findings should therefore be interpreted as a same-cohort comparison of discrimination for long-term all-cause mortality, rather than evidence that CORE-PH replaces established risk tools or provides superior treatment guidance. Using censoring-aware time-dependent analyses, CORE-PH demonstrated higher discrimination than COMPERA 2.0 at 36 and 60 months, but not at 12 months, whereas differences versus REVEAL Lite 2 were not statistically significant at any evaluated horizon. Accordingly, the present results do not support a generalized claim of superiority over established risk tools. The observed differences may partly reflect the complementary information provided by RV–PA interaction, systemic oxygen balance, and nutritional reserve, but may also reflect cohort-specific optimization because the components and thresholds were developed and evaluated in the same population.

The marked separation of CORE-PH risk categories, together with optimism-corrected discrimination and favorable apparent 36-month calibration, supports further evaluation of this multidomain approach. Clinically, the score may be most informative at the index diagnostic assessment, when invasive hemodynamics, a complete echocardiographic evaluation, and baseline laboratory data are available concurrently. Its potential role would not be to relabel patients who already demonstrate overt high-risk hemodynamics, but to identify individuals in whom adverse changes across several reserve domains coexist before a single conventional marker reaches its most severe range. This hypothesis requires prospective testing. Future studies should externally validate the score in larger multicenter cohorts, evaluate continuous and simplified clinically rounded thresholds, determine incremental value beyond existing risk strategies, externally validate time-dependent discrimination at clinically relevant horizons, and examine whether serial changes in CORE-PH track treatment response and subsequent outcomes.

### Limitations

Several limitations should be acknowledged. First, this was a retrospective, single-center study with a relatively small sample and 51 mortality events. Although the event count permitted a parsimonious multivariable model and bootstrap resampling demonstrated measurable optimism, internal validation cannot substitute for independent external validation. Importantly, the bootstrap procedure repeated the principal reproducible data-driven steps of threshold optimization, score reconstruction, and model refitting; however, the clinically selected component domains, equal-weight design choice, and derivation-cohort risk-group boundaries were not reselected using automated algorithms because no such algorithms were used in the original score-development process. Residual optimism related to these model-building decisions may therefore remain, further reinforcing the need for external validation. The initial screening population was substantially larger than the final analytical cohort, and the exclusion of patients with incomplete catheterization, echocardiographic, or laboratory data may have introduced selection bias. Comparisons between included patients and otherwise eligible patients excluded because of missing data were not available.

Second, CORE-PH was derived, thresholded, weighted, and evaluated in the same cohort. The apparent discriminatory performance may therefore overestimate performance in new populations. The three thresholds were selected according to cohort-specific mortality discrimination, and their exact decimal values should not be considered universal physiological boundaries. Equal weighting was chosen because the adjusted effect estimates were of broadly comparable magnitude and because a one-point structure improved bedside usability; however, the optimal weighting strategy remains uncertain. External validation should test both the original thresholds and clinically rounded alternatives and should reassess whether equal weighting remains appropriate.

Third, although conventional binary ROC analysis with Youden-index optimization was used to derive the three CORE-PH component thresholds, final comparative discrimination was assessed using censoring-aware time-dependent AUCs at prespecified 12-, 36-, and 60-month horizons. The threshold derivation nevertheless remains susceptible to same-cohort optimization, despite bootstrap internal validation incorporating repeated threshold re-estimation and score reconstruction. Moreover, the time-dependent comparisons remain internal to the derivation cohort and therefore require confirmation in independent populations. REVEAL Lite 2 and COMPERA 2.0 were developed for partly different clinical purposes and time horizons; their performance in the present study should therefore be interpreted as comparative discrimination within this cohort rather than validation or recalibration of their original risk estimates.

Fourth, the cohort was composed predominantly of patients with group 1 PAH, while only seven patients had group 4 CTEPH. The results should therefore be interpreted primarily as PAH findings and cannot establish the validity of CORE-PH in CTEPH. The physiological determinants of mixed venous oxygenation may differ between PAH and CTEPH, particularly because ventilation–perfusion mismatch may exert a greater influence in CTEPH. Etiology-specific validation is required, including separate analyses of idiopathic or heritable PAH, connective tissue disease-associated PAH, congenital heart disease-associated PAH, and CTEPH.

Fifth, the inclusion period extended from 2011 to 2025, during which diagnostic criteria, treatment availability, combination-treatment strategies, and referral practices evolved. Although all variables were obtained before PAH-specific therapy at the index evaluation, subsequent management was not standardized and may have influenced long-term survival. Although an additional sensitivity analysis incorporating index calendar year supported the robustness of the CORE-PH association, calendar year is only a proxy for treatment era and cannot fully account for residual temporal changes in treatment patterns, referral practices, and subsequent management over the study period.

Sixth, each CORE-PH component has measurement-related limitations. TAPSE is angle- and region-dependent, and estimated sPAP may be affected by tricuspid regurgitant signal quality, right atrial pressure estimation, severe tricuspid regurgitation, or advanced right ventricular failure. SvO_2_ is influenced by hemoglobin, arterial oxygenation, metabolic demand, oxygen supplementation, sampling conditions, and shunt anatomy. Although systematic caval sampling reduced contamination from intracardiac admixture, the use of inferior vena caval saturation in the two patients with anomalous pulmonary venous return introduced some methodological heterogeneity. PNI may be affected by inflammation, hepatic or renal dysfunction, infection, fluid status, and hematological conditions and should not be regarded as a specific measure of malnutrition. In addition, cardiac output was derived using estimated rather than directly measured oxygen consumption. Because assumed oxygen consumption may produce substantial individual error, calculated cardiac output and PVR—and consequently PVR-based hemodynamic classification in patients close to diagnostic thresholds—may have been affected. Although the persistence of the CORE-PH association in the stringent sensitivity cohort restricted to PVR >3 Wood units reduces concern that the principal findings were driven solely by borderline PVR classification, it does not eliminate measurement error inherent to the estimated-Fick approach [12].

Finally, only baseline measurements were analyzed. The study cannot determine whether CORE-PH identifies deterioration earlier than established tools, whether changes in the score reflect treatment response, or whether score-guided treatment improves outcomes. The primary endpoint was all-cause mortality, and potential differences in cause-specific mortality could not be evaluated. These considerations position CORE-PH as an internally validated, hypothesis-generating prognostic model that requires prospective and external confirmation before clinical implementation.

## 5. Conclusions

CORE-PH integrates three complementary dimensions of physiological reserve—right ventricular adaptation to pulmonary arterial load, systemic oxygen balance, and nutritional–immunological status—using variables routinely available during the initial diagnostic evaluation of patients with pulmonary arterial hypertension. In this predominantly PAH, treatment-naïve cohort, increasing CORE-PH scores were associated with a marked gradient in long-term mortality and demonstrated favorable time-dependent discrimination. Comparative performance varied according to the established risk tool and time horizon, with higher discrimination than COMPERA 2.0 at 36 and 60 months but no statistically significant superiority over REVEAL Lite 2 at the evaluated horizons. These findings support the concept that prognosis in PAH may be influenced not only by pulmonary vascular disease severity, but also by the cumulative loss of mechanical, oxygen-balance, and systemic reserve. CORE-PH should presently be regarded as a complementary and hypothesis-generating framework for PAH rather than a replacement for established risk tools, pending external validation across PAH populations and evaluation of its responsiveness to treatment. Its applicability to other forms of precapillary pulmonary hypertension, including CTEPH, remains uncertain and requires dedicated validation.

## Figures and Tables

**Table 3:** Association of the CORE-PH score and risk categories with all-cause mortality.

CORE-PH Measure	Univariable HR (95% CI)	*p* Value	Adjusted HR (95% CI)	*p* Value
CORE-PH, per 1-point increase	2.865 (2.005–4.096)	<0.001	2.645 (1.823–3.837)	<0.001
CORE-PH risk category		<0.001		<0.001
Low risk, 0–1 points	Reference		Reference	
Intermediate risk, 2 points	5.295 (2.403–11.666)	<0.001	4.359 (1.937–9.811)	<0.001
High risk, 3 points	11.000 (4.665–25.939)	<0.001	9.233 (3.849–22.144)	<0.001

Adjusted models included age and WHO-FC III–IV. CORE-PH was analyzed separately as a continuous score and as derivation-defined risk categories. Low risk was defined as 0–1 points, intermediate risk as 2 points, and high risk as 3 points. Abbreviations: CI, confidence interval; HR, hazard ratio; WHO-FC, World Health Organization functional class.

**Table 4:** Time-dependent discrimination of CORE-PH, COMPERA 2.0, and REVEAL Lite 2 for all-cause mortality in the primary cohort.

Time Horizon	CORE-PH AUC (95% CI)	COMPERA 2.0 AUC (95% CI)	*p* vs. CORE-PH	REVEAL Lite 2 AUC (95% CI)	*p* vs. CORE-PH
12 months	0.779 (0.663–0.894)	0.654 (0.470–0.838)	0.279	0.664 (0.460–0.869)	0.350
36 months	0.779 (0.683–0.875)	0.593 (0.448–0.738)	0.032	0.612 (0.460–0.765)	0.057
60 months	0.817 (0.738–0.896)	0.660 (0.543–0.777)	0.021	0.707 (0.587–0.827)	0.106

Time-dependent AUCs were estimated in the primary cohort (*n* = 105) using marginal inverse-probability-of-censoring weighting. Pairwise comparisons were performed between CORE-PH and each comparator at each prespecified time horizon. AUC, area under the curve; CI, confidence interval.

**Table 5:** Bootstrap internal validation of CORE-PH incorporating repeated threshold optimization and score reconstruction.

Performance Measure	Apparent Estimate	Estimated Optimism	Optimism-Corrected Estimate
Harrell’s C-index	0.747	0.028	0.719
Calibration slope	1.000	0.112	0.888

Internal validation was performed using 1000 bootstrap resamples incorporating repeated threshold optimization and score reconstruction. The apparent calibration slope is 1.000 by construction. Estimates are presented to three decimal places.

## Data Availability

The datasets generated and/or analyzed during the current study are not publicly available due to ethical and privacy restrictions but are available from the corresponding author on reasonable request and with permission of the Institutional Ethics Committee.

## Supplementary Materials

The following supporting information can be downloaded at: https://www.mdpi.com/article/10.3390/diagnostics16183057/s1, Supplementary Methods S1: Cardiac catheterization, oximetry, and hemodynamic calculations; Figure S1: Decision curve analyses comparing CORE-PH, COMPERA 2.0, and REVEAL Lite 2 for prediction of 12-month (A), 36-month (B), and 60-month (C) all-cause mortality. CORE-PH demonstrated greater apparent net benefit than the established risk tools across most clinically relevant low-to-moderate threshold probabilities, particularly at 36 and 60 months. The treat-all and treat-none strategies are shown for reference.

## Abbreviation

The following abbreviations are used in this manuscript:

6MWD	Six-minute walk distance
AUC	Area under the receiver operating characteristic curve
BMI	Body mass index
BNP	B-type natriuretic peptide
CI	Confidence interval
COMPERA	Comparative, Prospective Registry of Newly Initiated Therapies for Pulmonary Hypertension
CONUT	Controlling Nutritional Status
CORE-PH	Coupling, Oxygen Saturation, and Nutritional Reserve Evaluation in Pulmonary Hypertension
CRP	C-reactive protein
CTEPH	Chronic thromboembolic pulmonary hypertension
Ees/Ea	End-systolic elastance-to-arterial elastance ratio
ESC/ERS	European Society of Cardiology/European Respiratory Society
GNRI	Geriatric Nutritional Risk Index
HDL	High-density lipoprotein
HR	Hazard ratio
ISHLT	International Society for Heart and Lung Transplantation
LDL	Low-density lipoprotein
LVEF	Left ventricular ejection fraction
mPAP	Mean pulmonary artery pressure
NT-proBNP	N-terminal pro-B-type natriuretic peptide
PAH	Pulmonary arterial hypertension
PAWP	Pulmonary artery wedge pressure
PDE5	Phosphodiesterase type 5
PH	Pulmonary hypertension
PNI	Prognostic Nutritional Index
PVR	Pulmonary vascular resistance
RAP	Right atrial pressure
REVEAL	Registry to Evaluate Early and Long-term Pulmonary Arterial Hypertension Disease Management
RHC	Right heart catheterization
ROC	Receiver operating characteristic
RV–PA	Right ventricular–pulmonary arterial
sPAP	Systolic pulmonary artery pressure
SvO_2_	Mixed venous oxygen saturation
TAPSE	Tricuspid annular plane systolic excursion
TR	Tricuspid regurgitation
TRIPOD+AI	Transparent Reporting of a Multivariable Prediction Model for Individual Prognosis or Diagnosis plus Artificial Intelligence
WHO	World Health Organization
WHO-FC	World Health Organization functional class
