# Supplementary material for: CORE-PH: A 3-Component Score for Long-Term Mortality Risk in a Pulmonary Arterial Hypertension Cohort
Source: Diagnostics (Basel). 2026 Sep 20;16(18):3057. doi: 10.3390/diagnostics16183057 (PMC13605615; doi:10.3390/diagnostics16183057)
Supplement: Supplementary file 1 [file diagnostics-16-03057-s001.zip › diagnostics-4525070-supplementary.pdf]

Supplementary Methods S1. Cardiac catheterization, oximetry, and hemodynamic calculations

Combined right- and left-heart catheterization was performed through femoral venous and arterial access according to the institutional catheterization protocol. Hemodynamic pressures and blood samples for oxygen saturation analysis were systematically obtained from the inferior vena cava, superior vena cava, upper, middle, and lower right atrium, right ventricle, pulmonary artery, left ventricle, and aorta. All measurements included in the study were obtained during the first eligible diagnostic catheterization performed before the initiation of pulmonary arterial hypertension-specific therapy.

A complete oximetry run was routinely performed to detect an oxygen saturation step-up and to evaluate the presence and anatomical level of intracardiac shunting. Mixed venous oxygen saturation (SvO_2) used in the present analyses was calculated from superior vena caval (SVC) and inferior vena caval (IVC) oxygen saturations according to the following formula:

$$SvO_2\ (\%) = [3 \times \text{SVC oxygen saturation } (\%) + \text{IVC oxygen saturation } (\%)] / 4$$

Both SVC and IVC samples were obtained before intracardiac admixture. Although pulmonary arterial oxygen saturation was measured as part of the complete oximetry run, it was not used as the study SvO_2 value. In the two patients with partial anomalous pulmonary venous return involving the superior vena cava or its tributaries, the SVC sample was considered potentially contaminated by oxygenated pulmonary venous blood; therefore, IVC oxygen saturation was used as the mixed venous estimate.

Cardiac output was calculated using the estimated Fick principle according to the following equation:

$$\text{Cardiac output (L/min)} = \text{estimated oxygen consumption (mL/min)} / [\text{arterial oxygen content} - \text{mixed venous oxygen content (mL/L)}]$$

Resting oxygen consumption was estimated as follows:

$$\text{Estimated oxygen consumption (mL/min)} = 125\ \text{mL/min/m}^2 \times \text{body surface area (m}^2\text{)}$$

Body surface area was calculated using the Du Bois formula:

$$\text{Body surface area (m}^2\text{)} = 0.007184 \times \text{height (cm)}^{0.725} \times \text{weight (kg)}^{0.425}$$

Blood oxygen content was calculated from hemoglobin concentration and oxygen saturation using the following equations:

$$\text{Arterial oxygen content (mL/L)} = 1.36 \times \text{hemoglobin (g/dL)} \times \text{arterial oxygen saturation} \times 10$$

$$\text{Mixed venous oxygen content (mL/L)} = 1.36 \times \text{hemoglobin (g/dL)} \times \text{mixed venous oxygen saturation} \times 10$$

For these oxygen-content equations, oxygen saturations were entered as decimal fractions. Accordingly, a saturation of 95% was entered as 0.95.

When oxygen saturations were entered as percentages, the equivalent simplified cardiac output equation was:

Cardiac output (L/min) = [125 × body surface area] / [0.136 × hemoglobin × (arterial oxygen saturation − mixed venous oxygen saturation)]

In this simplified equation, arterial and mixed venous oxygen saturations were entered as percentages.

Cardiac index was calculated as follows:

Cardiac index (L/min/m²) = cardiac output (L/min) / body surface area (m²)

Pulmonary vascular resistance was calculated as follows:

Pulmonary vascular resistance (Wood units) = [mean pulmonary artery pressure (mmHg) − pulmonary artery wedge pressure (mmHg)] / cardiac output (L/min)

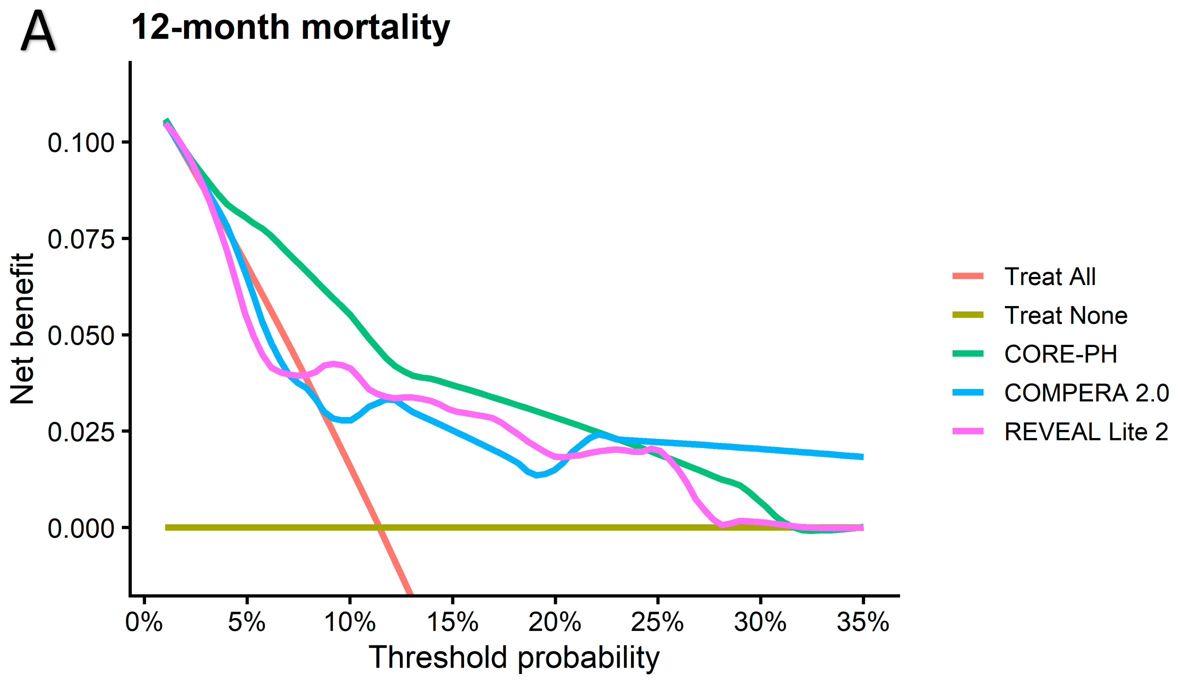

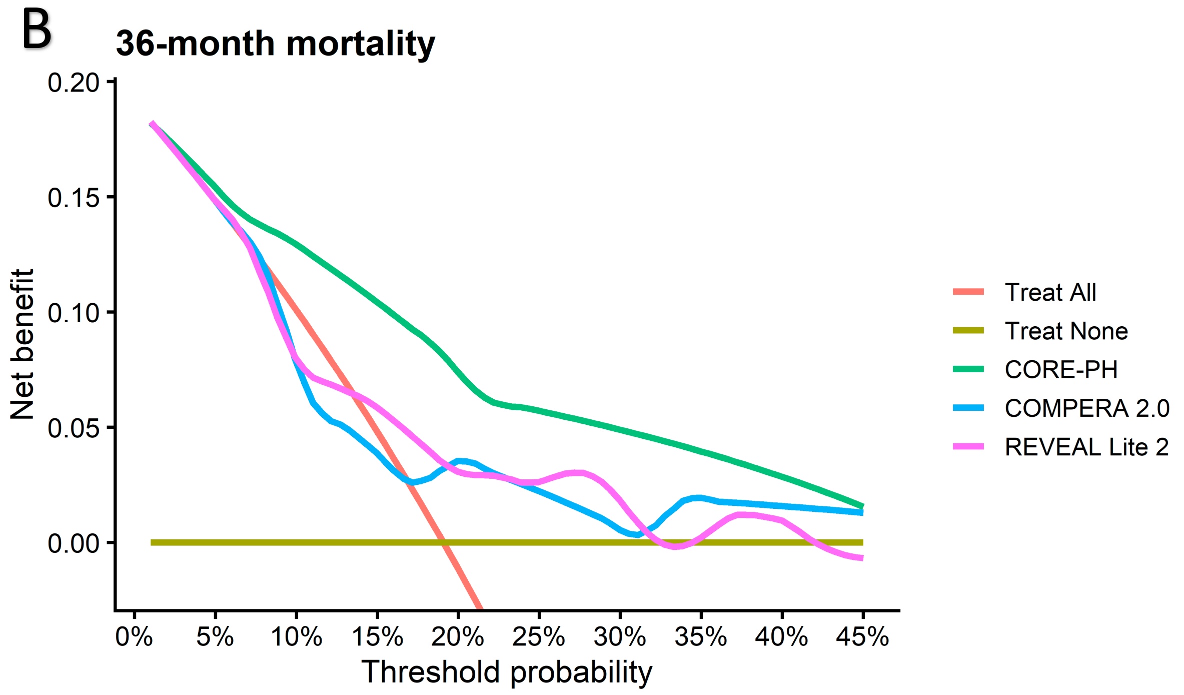

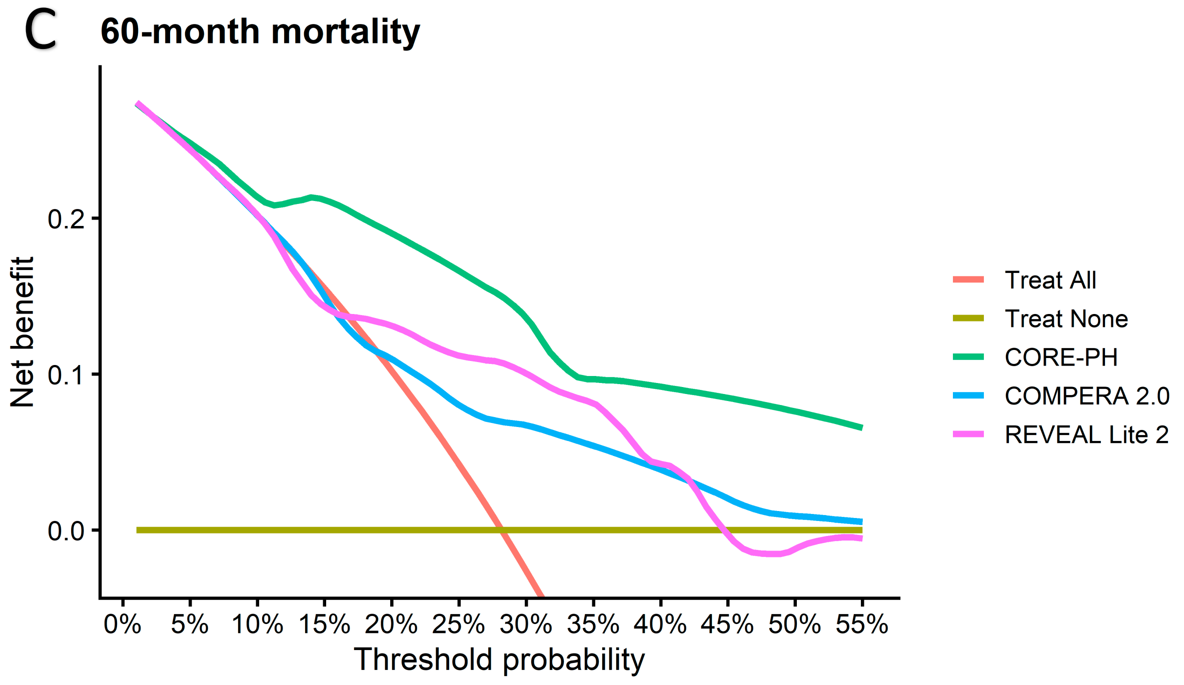

Supplementary Figure S1. Decision curve analyses comparing CORE-PH, COMPERA 2.0, and REVEAL Lite 2 for prediction of 12-month (A), 36-month (B), and 60-month (C) all-cause mortality. CORE-PH demonstrated greater apparent net benefit than the established risk tools across most clinically relevant low-to-moderate threshold probabilities, particularly at 36 and 60 months. The treat-all and treat-none strategies are shown for reference.
